# The Use of Internet-Based Health and Care Services by Elderly People in Europe and the Importance of the Country Context: Multilevel Study

**DOI:** 10.2196/15491

**Published:** 2020-06-03

**Authors:** Sebastian Merkel, Moritz Hess

**Affiliations:** 1 Faculty of Social Sciences Ruhr-University Bochum Bochum Germany; 2 SOCIUM Research Center on Inequality and Social Policy Bremen Germany

**Keywords:** eHealth, Europe, elderly people

## Abstract

**Background:**

Digital health care is becoming increasingly important, but it has the risk of further increasing the digital divide, as not all individuals have the opportunity, skills, and knowledge to fully benefit from potential advantages. In particular, elderly people have less experience with the internet, and hence, they are in danger of being excluded. Knowledge on the influences of the adoption of internet-based health and care services by elderly people will help to develop and promote strategies for decreasing the digital divide.

**Objective:**

This study examined if and how elderly people are using digital services to access health and social care. Moreover, it examined what personal characteristics are associated with using these services and if there are country differences.

**Methods:**

Data for this study were obtained from the Special Eurobarometer 460 (SB 460), which collected data on Europeans’ handling of and attitudes toward digital technologies, robots, and artificial intelligence, including data on the use of internet-based health and social care services, among 27,901 EU citizens aged 15 years or older. Multilevel logistic regression models were adopted to analyze the association of using the internet for health and social care services with several individual and country-level variables.

**Results:**

At the individual level, young age, high education, high social class, and living in an urban area were positively associated with a high probability of using internet-based health and social services. At the country level, the proportion of elderly people who participated in any training activity within the last month was positively associated with the proportion of elderly people using these services.

**Conclusions:**

The probability of using internet-based health and social services and their accompanying advantages strongly depend on the socioeconomic background. Training and educational programs might be helpful to mitigate these differences.

## Introduction

Health care systems in Europe and beyond are currently under pressure. Considering financial, demographic, and epidemiological developments, there is a need for new approaches to deliver health care equally and cost effectively and with the best medical outcomes [1]. There are many hopes on technological solutions, in particular digital technology, which promises to deliver health care without restrictions in time and space and has the potential to transform health care systems and the health care industry. With this technology, health information is obtained over the internet, vital signs are measured using smart devices and are directly sent to care providers, drugs are ordered over the internet, physicians are consulted from home, smartphone apps are used to manage chronic conditions, etc. Digital health care is an umbrella term for multiple buzzwords, including concepts like electronic health (eHealth), mobile health (mHealth), telemedicine, teleHealth, and many more. It can be defined as “the cultural transformation of how disruptive technologies that provide digital and objective data accessible to both caregivers and patients lead to an equal level doctor-patient relationship with shared decision-making and the democratization of care” [2].

Despite the potentials of digital health care, there are risks that lead to several challenges. In particular, the promise that all individuals will benefit equally needs to be questioned, as digital health requires not only infrastructure to use the internet, but also skills to operate digital technology [3,4]. Both, however, are unequally distributed across the population [5,6]. In this regard, a group of particular interest is elderly people. As decreasing fertility rates and increasing life expectancy are leading to demographic aging in North America, parts of Asia, and Europe, the absolute and relative numbers of old and very old (80 years or above) people are steadily increasing [7]. In addition, the elderly population is in particular need of health care and the possibilities and chances of digital health care for elderly people are particularly high [8]. However, there is a substantial part of the elderly population that does not use the internet, which is a precondition for using web-based health and social services.

When investigating influences on internet use for health and care services among elderly people, the following three aspects need to be considered: (1) factors influencing internet use in general; (2) factors influencing internet use for health-related purposes; and (3) factors influencing the capability to understand and process information, so-called eHealth literacy [3] or digital health literacy, which covers a set of skills to “search, select, appraise, and apply online health information” [4].

With regard to the first aspect, several studies reported that internet use declines with increasing age in Western societies [9,10]. Eurostat data for 2018 showed that 98% of EU-28 citizens aged 16 to 24 years used the internet within the last 12 months, but only 78% of those aged 55 to 64 years and 48% of those aged 65 to 74 years used the internet within the last 12 months [11]. There are differences between countries. Although the percentages have increased over the years, the use of internet technology by elderly people has declined with increasing age. This decline can be explained by several factors at not only the individual level, but also the “meso” and “macro” levels. At the individual level, factors, such as education [9,10,12] and income [9,10,13], are associated with digital divide. Moreover, male sex is associated with higher internet use, and age 65 years or above [10], health [13,14], and experience with computers during working life have an effect on internet use in old age [6,15]. At the “meso” level, social support is positively associated with internet use in old age. Those with a strong social network are more likely to use the internet, as they make use of internet and communication technologies to curate their network; in addition, individuals with a large social network are more likely to be introduced to new technologies [16,17]. Moreover, support programs aimed at introducing elderly people to the internet have an effect [18]. At the “macro” level, several studies have shown a link between infrastructure and internet use. As individuals in rural areas often have less access to broadband or mobile connections, they are less likely to use the internet [10]. Another aspect that needs to be mentioned here is technical socialization. According to the “technology generation theory” [19], birth cohorts differ according to the technological devices they have used while growing up.

We were interested in exploring eHealth use in terms of using the internet to access health and social care services among those who were already on the internet. Considering the use of the internet for health purposes, previous research has revealed multiple influences on the use of new digital technologies to access health care by elderly people. There are, however, multiple overlapping factors. As for internet use in general, sociodemographic characteristics, such as gender (women are more likely to use the internet for health and social care than men), age, education, and household income, are associated with using the internet for health and social care services [20-25]. At the “meso” level, social networks are reported to have a positive effect [24]. At the “macro” level, previous results found that individuals in rural areas seem to use eHealth less often than those in more densely populated areas [24]. Although we did not encounter studies investigating the effect of the country context on the internet-based use of health and social care services, we assume that it has an influence. We assume that life-long learning programs have a comparable effect on eHealth use as on internet use in general. In rich countries, we hypothesize a high proportion of eHealth users and a high number of elderly people with the resources to access eHealth. The share of the national budget spent for elderly people is positively associated with eHealth use among elderly people, as more financial resources are provided. In addition, we hypothesize that in countries with a high proportion of elderly people, these elderly people represent a large group of customers for providers of eHealth and hence are a target for advertisements. Finally, as good access to the internet is a necessary condition to use digital health and social care services, we assume that the proportion of elderly people who use these services increases with an increase in a country’s quality of internet access.

It is important to determine if and how elderly people use the internet to access health care; what personal characteristics are associated with using eHealth; and whether there are country differences in access to eHealth, and if so, how can these be explained. To obtain this information, this study analyzed data from a Special Eurobarometer [26], using multilevel logistic regression. It investigated how many people in Europe use digital health care services. Furthermore, it explored which variables at the individual level and the country level are associated with a high probability of the use of digital health care services. Controlling for age, employment status, marital status, and self-perceived class, the study hypothesized that elderly women are more likely to use the internet to access health and social care (H1), elderly people with a high education level are more likely to use eHealth (H2), elderly people living in urban areas are more likely to use eHealth (H3), and elderly people living alone are less likely to use eHealth (H4). At the country level, it hypothesized that elderly people in countries where life-long learning is more common are more likely to use eHealth (H5), elderly people in rich countries are more likely to use eHealth (H6), elderly people in countries where a large share of the welfare state’s budget is spent on the elderly population are more likely to use eHealth (H7), elderly people in countries where demographic ageing is more developed are more likely to use eHealth (H8), and elderly people in countries where access to the internet is good are more likely to use eHealth (H9).

This study contributes to the field in several ways. First, the inclusion of individual as well as country level determinants of the probability of using digital health care services provides a more holistic picture of the potential of digitalization for health care among elderly people. The findings shed light on relevant disparities in the use of digital health care services among elderly people at the individual and country levels. The second contribution is the data used in the study, which were derived from a recent survey conducted in 2017. As digital technologies are changing quickly and new possibilities for digital health care provision are being developed constantly, regular monitoring of how elderly people use this approach is necessary. The third contribution is the comparative perspective. The inclusion of several countries in the analysis allows the identification of factors that foster and hinder the use of eHealth, which can be transformed into policy recommendations.

## Methods

### Data and Sample

The analysis in this study was conducted with data derived from the Special Eurobarometer 460 (SB 460) *Attitudes toward the impact of digitization and automation*, which collected data on Europeans’ handling of and attitudes toward digital technologies, robots, and artificial intelligence, including questions on the use of internet-based health care in the year 2017. The SB 460 is part of the Eurobarometer program that includes several public opinion surveys among the citizens of the European Union on a variety of topics. For the SB 460, the TNS Political & Social network performed face-to-face interviews for 27,901 EU citizens aged 15 years or older. The interviews took place at the home of the interviewees and in their native language. Sampling was performed with a multistage random probability approach [26]. For the analysis of this study, the sample was restricted to adults aged 65 years or older who in general use the internet, which resulted in a sample size of 6900. In addition, it has the advantage of sufficient observational units (countries) at the upper level to conduct multilevel regression analyses.

### Analysis Strategy

Multilevel logistic regression models were used to analyze the association of using the internet for health care services with several individual and country-level variables. Multilevel regression is an adequate tool of analysis when the data have a hierarchical structure with units at the lower level nested in those at the higher level [27]; in this analysis, individual respondents nested in countries. However, as the data had a cross-sectional nature, no causal but only correlational relations can be derived from the results. The analysis was conducted using Stata 14 (StataCorp).

### Measures

The variable for the use of digital health care services was based on the following question: *In the last 12 months, how often have you used, if ever, health and care services provided over the internet without having to go to the hospital or doctor's surgery (for example, by getting a prescription or a consultation online)?* The respondents could answer this question with any one of the following four predefined statements: *once*, *twice*, *thrice or more*, and *never.* Missing data were negligible (<1%, n=16). As over 83.87% (5787/6900) of the respondents reported never using internet-based health care services, the other three categories (once, twice, and thrice or more) were summarized into one category. This resulted in the dichotomous variable “use of digital health care services,” with values of yes and no.

At the individual level, age, gender, education, social class, marital status, employment status, and urbanization degree were correlated with the use of digital health care services. Education was measured according to age on completion of education and was divided into the following three categories: younger than 15 years, 15 to 20 years, and older than 20 years. Social class was divided into the following three categories: high, medium, and low. Marital status was dichotomized into having a partner and not having a partner. Additionally, employment status was dichotomized into being employed and not being employed. Information regarding urbanization degree had the following three categories: rural area, towns and suburbs, and cities.

At the country level, we included five variables. The proportion of elderly people (aged 65 years or older) who had participated in educational or training activities within the last 4 weeks was used as a measurement for common life-long learning among elderly people. The gross domestic product per person was used to measure the economic development of the countries. To measure the spending for old age, we included the share of the national budget that was used for elderly people. The proportion of people older than 64 years to people younger than 65 years was used to measure how far demographic ageing in a country has progressed. Finally, the subdimension connectivity of the Digital Economy and Society Index was used to measure a country’s access to the internet. Data for all five indicators were derived from Eurostat, the statistical office of the European Union [28].

## Results

### Descriptive Results

**Figure 1:**
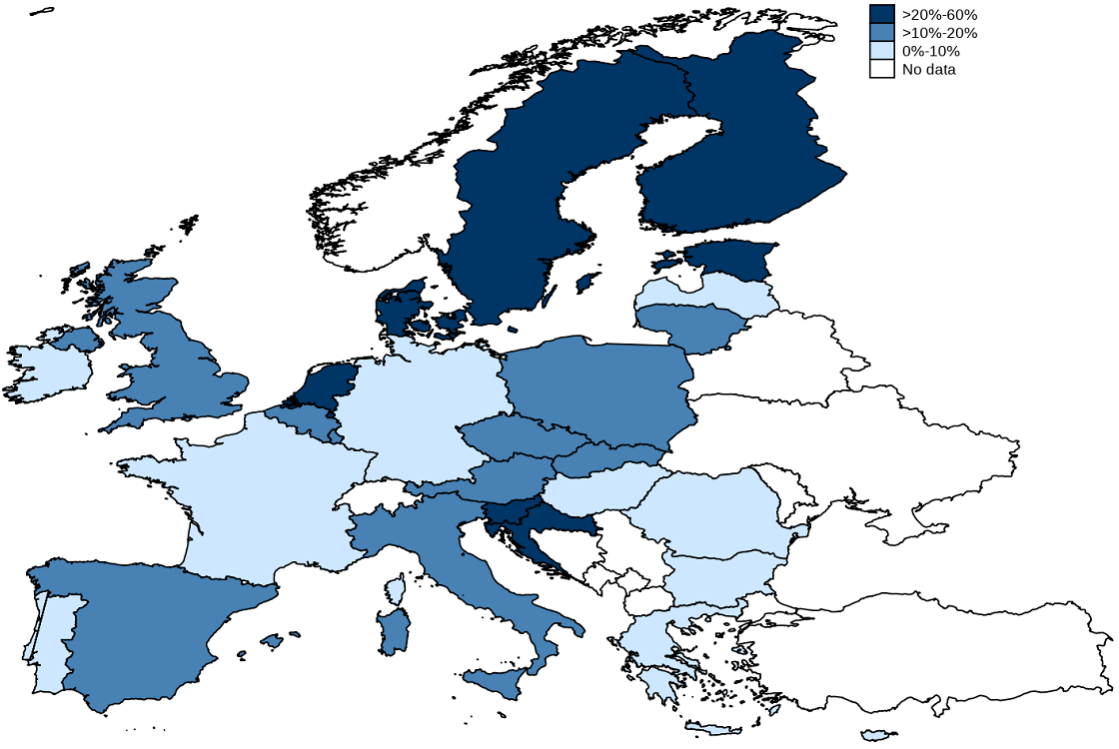
Geographical distribution of elderly people using internet-based health care services.

Figure 1 and Table 1 show how many elderly people are using internet-based health care services in different European countries. The highest rates were found in Scandinavian countries and Estonia, and the lowest rates were in Malta, Cyprus, and Germany.

**Table 1:** Use of internet-based health care services among elderly people (aged 65 years or older, N=6900).

Country	Users, n (%)	Nonusers, n (%)
Austria	14 (8.3)	176 (91.7)
Belgium	34 (11.8)	273 (88.2)
Bulgaria	11 (7.5)	149 (92.5)
Croatia	31 (25.8)	89 (74.2)
Cyprus	2 (2.5)	117 (97.5)
Czech Republic	14 (6.8)	183 (93.2)
Denmark	119 (38.9)	187 (61.1)
Estonia	126 (43.8)	161 (56.2)
Finland	144 (43.9)	187 (56.1)
France	13 (4.6)	273 (95.4)
Germany	24 (4.9)	491 (95.1)
Greece	11 (5.7)	176 (94.3)
Hungary	14 (4.6)	272 (95.4)
Ireland	9 (4.3)	206 (95.7)
Italy	23 (12.4)	166 (87.6)
Latvia	14 (6.9)	200 (93.1)
Lithuania	38 (11.2)	317 (88.8)
Luxembourg	22 (18.2)	100 (81.8)
Malta	7 (4.5)	157 (95.5)
Netherlands	78 (28.7)	200 (71.3)
Poland	30 (14.7)	165 (85.3)
Portugal	15 (8.0)	176 (92.0)
Romania	11 (10.5)	105 (89.5)
Slovakia	53 (21.6)	183 (78.4)
Slovenia	73 (26.7)	196 (73.3)
Spain	23 (10.3)	197 (89.7)
Sweden	103 (22.9)	338 (77.1)
United Kingdom	57 (14.5)	347 (85.5)

On comparing users and nonusers of eHealth (Table 2), users were on average younger (71.96 vs 73.04 years) and better educated (proportion of high education: 559/1113, 50.22% vs 1582/5787, 27.34%). In particular, among those from a high social class, there was a higher proportion of users than nonusers (162/1113, 14.56% vs 374/5787, 6.46%). Additionally, among those in employment and with a partner, there was a higher proportion of users than nonusers (95/1113, 8.54% vs 324/5787, 5.60% and 684/1113, 61.46% vs 3152/5787, 54.47%, respectively). Among those in one-person households, there was a lower proportion of users than nonusers (366/1113, 32.88% vs 2246/5787, 38.82%).

**Table 2:** Characteristics of the users and nonusers of internet-based health care services.

Characteristic		Nonusers (N=5787), mean or n (%)	Users (N=1113), mean or n (%)	*P* value
Age (years)		73.04	71.96	.00^a^
**Gender**				.22^b^
	Male	2676 (46.24)	536 (48.25)	
	Female	3111 (53.76)	576 (51.75)	
**Age when education was completed (years)**				.00^b^
	<16	1855 (32.05)	153 (13.75)	
	16-19	2350 (40.61)	401 (36.03)	
	≥20	1582 (27.34)	559 (50.22)	
**Social class**				.00^b^
	Low	2868 (49.56)	354 (31.81)	
	Medium	2545 (42.98)	597 (53.64)	
	High	374 (6.46)	162 (14.56)	
**Employment status**				.00^b^
	Employed	324 (5.60)	95 (8.54)	
	Not employed	5463 (94.40)	1018 (91.46)	
**Marital status**				.00^b^
	With partner	3152 (54.47)	684 (61.46)	
	Without partner	2635 (45.53)	429 (38.54)	
**Household size**				.00^b^
	One	2246 (38.82)	366 (32.88)	
	Two	3024 (52.26)	673 (60.47)	
	Three	345 (5.96)	45 (4.04)	
	Four or more	171 (2.96)	29 (2.61)	
**Population density**				.09^b^
	Rural area	1673 (28.91)	286 (25.70)	
	Towns and suburbs	1974 (34.11)	390 (35.04)	
	Cities	2140 (36.98)	437 (39.26)	

^a^*t* test.

^b^Chi square test.

The results from the multivariate analysis are presented in Table 3. The intraclass correlation of >0.16 shows that a substantial part of the dependent variable’s variation was at the country level and the use of multilevel models is appropriate. At the individual level, the regression found no relevant association between gender and internet use for health and social care. Higher age was associated with less likelihood of using eHealth (OR 0.97, 95% CI 0.96-0.98, *P*<.001). The results also showed significant positive associations between education (16-19 years: OR 1.43, 95% CI 1.15-2.79, *P*<.001; ≥20 years: OR 1.95, 95% CI 1.54-2.46, *P*<.001) and social class (medium: OR 1.45, 95% CI 1.23-1.71, *P*<.001; high: OR 2.00, 95% CI 1.53-2.61, *P*<.001) on one hand and use of eHealth on the other. Employment status, marital status, and household size were not associated with eHealth use. Population density was associated positively with eHealth use (cities: OR 1.23, 95% CI 1.02-1.48, *P*=.03). At the country level, only the proportion of elderly people who participated in educational activities was significantly associated with eHealth use (OR 1.06, 95% CI 1.01-1.13, *P*=.02).

**Table 3:** Regression findings regarding the use of internet-based health care services.

Variable			Model (N=6899)												
			M1		M2			M3			M4			M5	
			OR (SE)	95% CI	OR (SE)	95% CI	OR (SE)		95% CI	OR (SE)		95% CI	OR (SE)		95% CI
**Individual-level variables**															
	Age		0.97^a^ (0.01)	0.96-0.98	0.97^a^ (0.01)	0.96-0.98	0.97^a^ (0.01)		0.96-0.98	0.97^a^ (0.01)		0.96-0.98	0.97^a^ (0.01)		0.96-0.98
	**Gender (ref^b^: male)**														
		Female	1.02 (0.08)	0.88-0.19	1.02 (0.08)	0.88-0.19	1.02 (0.08)		0.88-0.19	1.02 (0.08)		0.88-0.19	1.02 (0.08)		0.88-0.19
	**Age (years) when education was completed (ref: <16)**														
		16-19	1.43^c^ (0.16)	1.15-2.79	1.43^c^ (0.16)	1.15-2.79	1.43^c^ (0.16)		1.15-2.79	1.43^c^ (0.16)		1.15-2.79	1.43^c^ (0.16)		1.15-2.79
		≥20	1.95^a^ (0.23)	1.54-2.46	1.95^a^ (0.23)	1.54-2.46	1.95^a^ (0.23)		1.54-2.46	1.95^a^ (0.23)		1.54-2.46	1.95^a^ (0.23)		1.54-2.46
	**Social class (ref: low)**														
		Medium	1.45^a^ (0.12)	1.23-1.71	1.45^a^ (0.12)	1.23-1.71	1.45^a^ (0.12)		1.23-1.71	1.45^a^ (0.12)		1.23-1.71	1.45^a^ (0.12)		1.23-1.71
		High	2.00^a^ (0.26)	1.53-2.61	2.00^a^ (0.26)	1.53-2.61	2.00^a^ (0.26)		1.53-2.61	2.00^a^ (0.26)		1.53-2.61	2.00^a^ (0.26)		1.53-2.61
	**Employment status (ref: employed)**														
		Not employed	0.92 (0.13)	0.70-1.21	0.92 (0.13)	0.70-1.21	0.92 (0.13)		0.70-1.21	0.92 (0.13)		0.70-1.21	0.92 (0.13)		0.70-1.21
	**Marital status (ref: with partner)**														
		Without partner	0.81 (0.10)	0.63-1.03	0.81 (0.10)	0.63-1.03	0.81 (0.10)		0.63-1.03	0.81 (0.10)		0.63-1.03	0.81 (0.10)		0.63-1.03
	**Household size (ref: one)**														
		Two	1.17 (0.15)	0.90-1.51	1.17 (0.15)	0.90-1.51	1.17 (0.15)		0.90-1.51	1.17 (0.15)		0.90-1.51	1.17 (0.15)		0.90-1.51
		Three	0.79 (0.16)	0.52-1.17	0.79 (0.16)	0.52-1.17	0.79 (0.16)		0.52-1.17	0.79 (0.16)		0.52-1.17	0.79 (0.16)		0.52-1.17
		Four or more	1.20 (0.29)	0.74-1.93	1.20 (0.29)	0.74-1.93	1.20 (0.29)		0.74-1.93	1.20 (0.29)		0.74-1.93	1.20 (0.29)		0.74-1.93
	**Population density (ref: rural area)**														
		Towns and suburbs	1.11 (0.11)	0.92-1.34	1.11 (0.11)	0.92-1.34	1.11 (0.11)		0.92-1.34	1.11 (0.11)		0.92-1.34	1.11 (0.11)		0.92-1.34
		Cities	1.23^d^ (0.12)	1.02-1.48	1.23^d^ (0.12)	1.02-1.48	1.23^d^ (0.12)		1.02-1.48	1.23^d^ (0.12)		1.02-1.48	1.23^d^ (0.12)		1.02-1.48
**Country-level variables**															
	Life-long learning (M1)		1.06^d^ (0.03)	1.01-1.13	N/A^e^	N/A	N/A		N/A	N/A		N/A	N/A		N/A
	GDP^f^ per person (M2)		N/A	N/A	1.00 (0.00)	0.99-1.00	N/A		N/A	N/A		N/A	N/A		N/A
	Spending for old age (M3)		N/A	N/A	N/A	N/A	1.03 (0.06)		0.90-1.17	N/A		N/A	N/A		N/A
	Old age ratio (M4)		N/A	N/A	N/A	N/A	N/A		N/A	1.03 (0.04)		0.94-1.12	N/A		N/A
	Connectivity (M5)		N/A	N/A	N/A	N/A	N/A		N/A	N/A		N/A	1.01 (0.01)		0.99-1.05
ICC^g^			0.16		0.18		0.18			0.18			0.17		

^a^*P*<.001.

^b^Ref: reference.

^c^*P*<.01.

^d^*P*<.05.

^e^N/A: not applicable.

^f^GDP: gross domestic product.

^g^ICC: intraclass correlation coefficient.

## Discussion

This study explored the determinants of internet-based use of health and care services among elderly Europeans, using data from the SB 460 and the multilevel regression technique. Our study focused on elderly people who were already using the internet and who went on the internet to use health and care services. We found that a large proportion of elderly people in Scandinavia and Estonia use the internet for health and care services and very few people in Malta, Cyprus, and Germany use the internet for these services. One explanation is the difference in broadband and mobile internet availability between these countries. Scandinavia and Estonia have a high number of households with internet access, whereas Malta, Cyprus, and Germany lag behind in terms of broadband availability [29]. Consequently, there is a need to offer proper infrastructure on a broad basis. Although the number of practicing physicians [30] does not seem to make a difference, the population density is comparably high in Malta, Cyprus, and Germany making it easier to access health and care services [31]. However, when living in rural areas, access to offline health care can be problematic and online services could help to make health and social care available even at long distances.

By analyzing the factors at the micro level, which seem to have an influence on the use of internet-based health and social care services among elderly people, we could confirm the results of previous research. In line with previous results, we found that users who are better educated and from a higher class are more likely to use these services [19]. We also found that elderly people in rural areas are less likely to use the internet for health and social services. This is in line with the results in the study by Torrent-Sellens et al, who used survey data from European citizens aged 16 to 74 years. Their data revealed that people living in less densely populated areas had a low propensity toward intensive eHealth use [24]. The results from the regression analyses showed that when controlling for potentially confounding variables, nonusers were older than users of internet-based health and care services. As shown by the results of other studies [9,10], age needs to be taken into account when analyzing eHealth use among populations. Several of our findings (those with high education are more likely to use eHealth, living in a city is positively associated with eHealth use, etc) seem to not apply to young cohorts.

In addition to scientific implications, political and societal implications can be drawn. The results underline several issues accompanying the spread of digital technology in general, but particularly in health care. Although internet-based health and care services have main advantages to support elderly people, policy makers and other stakeholders should also acknowledge that most elderly people do not use these services. Additionally, the probability to use these services does correlate with socioeconomic status and place of living. In particular, people with a low socioeconomic status and those living in rural areas seem to be at risk of being excluded from chances to use eHealth, although, in particular, the latter group could benefit from remote health services. This has the risk of increasing social inequality. Technology can cause or intensify social inequality and ultimately lead to social exclusion. Against this background, the capability of using modern technology itself can be seen as a dimension of social inequality [32]. Previous research has shown that people going on the internet for health services experienced improved outcomes with respect to their knowledge of health issues, health communication with medical professionals, decision-making about their health issues, and proper use of health services [23]. Consequently, this could lead to additional inequalities. As eHealth solutions are pushed at the national and EU level, policymakers should acknowledge these differences. The fact that elderly people often do not use the internet can itself be seen as one reason for the relatively low diffusion of eHealth in several countries [33]. Consequently, training and educational programs on how to use digital technologies in general and eHealth services in particular can support these elderly people who have little or no experience with eHealth. This argument is supported by the finding of this study that the proportion of elderly people participating in further education at the country level and the use of eHealth are closely related.

Our study has several limitations. First, it included a specific database. The Special Eurobarometer only includes one item that asks about eHealth (going on the internet to use health and care services). Hence, we could not draw any conclusions on the different facets of eHealth and could not provide detailed information on patterns of use. Second, we only investigated people who were using the internet and did not cover those not using the internet. We were mainly interested in exploring the personal characteristics of those using internet-based health and care services and macro factors potentially influencing the use. This leaves room for a more detailed analysis including those not using the internet. Although we could confirm most of the results of previous studies and add new aspects to the discussion on the use of digital health by elderly people, there were several limitations. Third, the data used for the analysis were cross-sectional data; hence, no causal links could be made between the different variables. Fourth, the analysis was limited to European countries; however, demographic ageing and digitalization are global trends.

Despite these limitations, this study contributes to the field in three ways. First, the inclusion of individual- and country-level determinants of the probability of using digital health care services provides a more holistic picture of the potential of digitalization for health care among elderly people. The findings shed light on the relevant disparities in the use of digital health care services among elderly people at the individual and country levels. Second, the data used in this study were derived from the most recent survey conducted in 2017. As digital technologies are changing at a fast pace and new possibilities for digital health care provision are being developed constantly, regular monitoring of how elderly people use these services is necessary. Third, there was a comparative perspective. The inclusion of several countries in the analysis allowed the identification of factors that foster and hinder the use of eHealth, which can be transformed into policy recommendations.

## Abbreviations

SB 460: Special Eurobarometer 460

## References

[1] Bengoa R (2013). Transforming health care: an approach to system-wide implementation. Int J Integr Care.

[2] Meskó B, Drobni Z, Bényei É, Gergely B, Győrffy Z (2017). Digital health is a cultural transformation of traditional healthcare. Mhealth.

[3] Norman CD, Skinner HA (2006). eHealth Literacy: Essential Skills for Consumer Health in a Networked World. J Med Internet Res.

[4] van der Vaart R, Drossaert C (2017). Development of the Digital Health Literacy Instrument: Measuring a Broad Spectrum of Health 1.0 and Health 2.0 Skills. J Med Internet Res.

[5] Mackert M, Mabry-Flynn A, Champlin S, Donovan EE, Pounders K (2016). Health Literacy and Health Information Technology Adoption: The Potential for a New Digital Divide. J Med Internet Res.

[6] Vulpe S, Crăciun A (2020). Silver surfers from a European perspective: technology communication usage among European seniors. Eur J Ageing.

[7] Harper S, Torp C (2015). The Challenges of Twenty-First-Century Demography. Challenges of Aging: Pensions, Retirement and Generational Justice.

[8] Melchiorre MG, Papa R, Rijken M, van Ginneken E, Hujala A, Barbabella F (2018). eHealth in integrated care programs for people with multimorbidity in Europe: Insights from the ICARE4EU project. Health Policy.

[9] Friemel TN (2014). The digital divide has grown old: Determinants of a digital divide among seniors. New Media & Society.

[10] König R, Seifert A, Doh M (2018). Internet use among older Europeans: an analysis based on SHARE data. Univ Access Inf Soc.

[11] (2019). Eurostat.

[12] Neves BB, Amaro F, Fonseca JR (2013). Coming of (Old) Age in the Digital Age: ICT Usage and Non-Usage among Older Adults. Sociological Research Online.

[13] Hargittai E, Piper A, Morris M (2018). From internet access to internet skills: digital inequality among older adults. Univ Access Inf Soc.

[14] Cresci M, Yarandi H, Morrell RW (2010). Pro-Nets Versus No-Nets: Differences in Urban Older Adults' Predilections for Internet Use. Educational Gerontology.

[15] Korupp S (2005). Causes and Trends of the Digital Divide. European Sociological Review.

[16] Heo J, Chun S, Lee S, Lee KH, Kim J (2015). Internet use and well-being in older adults. Cyberpsychol Behav Soc Netw.

[17] Barbosa Neves B, Fonseca JR, Amaro F, Pasqualotti A (2018). Social capital and Internet use in an age-comparative perspective with a focus on later life. PLoS One.

[18] Bertera EM, Bertera RL, Morgan R, Wuertz E, Attey AM (2007). Training Older Adults to Access Health Information. Educational Gerontology.

[19] Sackmann R, Winkler O (2013). Technology generations revisited: The internet generation. Gerontechnology.

[20] Rice RE (2006). Influences, usage, and outcomes of Internet health information searching: multivariate results from the Pew surveys. Int J Med Inform.

[21] Choi NG, Dinitto DM (2013). The digital divide among low-income homebound older adults: Internet use patterns, eHealth literacy, and attitudes toward computer/Internet use. J Med Internet Res.

[22] Gordon NP, Hornbrook MC (2018). Older adults' readiness to engage with eHealth patient education and self-care resources: a cross-sectional survey. BMC Health Serv Res.

[23] Yoon H, Jang Y, Vaughan PW, Garcia M (2020). Older Adults' Internet Use for Health Information: Digital Divide by Race/Ethnicity and Socioeconomic Status. J Appl Gerontol.

[24] Torrent-Sellens J, Díaz-Chao Á, Soler-Ramos I, Saigí-Rubió F (2016). Modelling and Predicting eHealth Usage in Europe: A Multidimensional Approach From an Online Survey of 13,000 European Union Internet Users. J Med Internet Res.

[25] Kontos E, Blake KD, Chou WS, Prestin A (2014). Predictors of eHealth usage: insights on the digital divide from the Health Information National Trends Survey 2012. J Med Internet Res.

[26] (2017). European Commission.

[27] Hox J, Moerbeek M, van de Schoot R (2018). Multilevel analysis: Techniques and applications.

[28] Eurostat.

[29] Eurostat (2019). https://ec.europa.eu/eurostat/databrowser/view/tin00073/default/table?lang=en.

[30] (2019). Eurostat.

[31] Eurostat (2019). Eurostat.

[32] Pelizäus-Hoffmeister H (2013). Zur Bedeutung von Technik im Alltag Älterer Theorie und Empirie aus soziologischer Perspektive.

[33] Romano MF, Sardella MV, Alboni F, Russo L, Mariotti R, Nicastro I, Barletta V, Di Bello V (2015). Is the digital divide an obstacle to e-health? An analysis of the situation in Europe and in Italy. Telemed J E Health.

